# Feasibility study of an insole-type active assist device for ankle alignment correction during stepping in patients with knee osteoarthritis

**DOI:** 10.1186/s42490-026-00104-z

**Published:** 2026-01-30

**Authors:** Taku Itami, Ryuichi Hirota, Masakatsu Iwase, Yoichi Oi, Koji Ebisu, Takaaki Aoki

**Affiliations:** 1 Department of Electronics and Bioinfomatics School of Science and Technology, Meiji University, 1-1-1 Higashi-Mita, Tama-ku, Kawasaki-shi, Kanagawa, 214-8571 Japan; 2 Department of Electrical and Electronic Engineering, Graduate School of Science and Engineering, Aoyama Gakuin University, Kanagawa, Japan; 3 AISIN Corporation, Aichi, Japan; 4 Department of Rehabilitation and Orthopedics, Gifu University, Gifu, Japan

**Keywords:** Insole-type device, Inversion-eversion, Ankle angle, Stepping

## Abstract

**Objectives:**

This feasibility study aimed to examine whether an insole-type active assist device designed to dynamically adjust ankle alignment at heel contact can be safely delivered and evaluated during an on-the-spot stepping task in patients with medial knee osteoarthritis (OA). The study specifically assessed the feasibility of intervention delivery, testing procedures, and motion-capture-based outcome measurement.

**Methods:**

Six ambulatory patients with medial knee OA (Kellgren–Lawrence grade II–III) performed repeated on-the-spot stepping trials under two conditions: Active (device control enabled) and Inactive (device control disabled). The assist device tilts the heel toward eversion in response to detected ankle inversion at heel contact. Feasibility outcomes included participant recruitment and completion, safe execution of the stepping task, device activation during trials, successful acquisition and analysis of motion capture data, and occurrence of adverse events. Lateral knee thrust was quantified descriptively using a three-dimensional motion capture system to characterize measurement variability and inform future study design.

**Results:**

All participants provided informed consent and completed the stepping protocol (6/6, 100%), with no adverse events observed. The stepping task and testing procedures were safely performed in all cases. Motion capture data were successfully acquired and analyzed for all trials (90/90, 100%). The assistive mechanism was activated in at least one stepping trial in five of six participants (83%), with activation occurring in 39 of 90 stepping trials (43%). Across conditions, lateral knee thrust values showed substantial inter-individual and condition-related variability, ranging approximately from 30 to 110 mm across participants.

**Conclusions:**

This study demonstrates the feasibility and safety of delivering an insole-type active assist intervention and conducting motion-capture-based evaluations during an on-the-spot stepping task in patients with medial knee OA. The observed variability in lateral knee thrust highlights important considerations for outcome selection and sample size planning, supporting progression to future adequately powered studies to evaluate clinical and biomechanical effectiveness.

## Introduction

Knee osteoarthritis (knee OA) is a prevalent musculoskeletal disorder among older adults and a major cause of impaired lower-limb function. In Japan, approximately 10 million individuals are estimated to have symptomatic knee OA, with nearly 30 million potential patients based on radiological diagnoses [1]. As the disease progresses, joint deformity, pain during movement, and restricted range of motion substantially limit standing and stepping-related activities, leading to reduced mobility and quality of life [2].

Several wearable and assistive robots have been developed to support lower-limb function in elderly individuals and persons with disabilities [3–5]. However, numerous challenges remain in the practical application of these devices, including physical constraints caused by limited joint mobility and muscle atrophy due to reliance on external power sources such as motors or pneumatic systems [6]. In contrast, insole-based interventions offer a compact and less intrusive approach for modifying lower-limb biomechanics. Previous studies on lateral wedge insoles (LWI) have demonstrated that altering heel alignment can laterally shift the center of pressure (COP), modify ankle and knee joint kinematics, and reduce medial knee joint loading [7]. Other research highlighted the effectiveness of insoles in improving lower-limb biomechanics [8]. However, because lower-limb kinematics vary from step to step, a static insole cannot continuously provide optimal alignment correction at critical events such as heel contact.

During heel contact, proper alignment of the heel is essential for effective load absorption by the ankle and knee joints. When the heel contacts the ground in a neutral position, impact forces can be distributed efficiently across the lower limb. In contrast, abnormal ankle posture at heel contact may induce instability, leading to altered lower-limb mechanics and increased mechanical stress during stance [9]. From a biomechanical perspective, excessive ankle inversion at heel contact causes the ground reaction force vector to pass medially relative to the knee joint center, thereby increasing the knee adduction moment (KAM) and contributing to the varus-thrust phenomenon commonly observed in patients with medial knee osteoarthritis. Actively guiding the ankle toward a neutral or slightly everted position at heel contact can laterally shift the COP, realign the load-bearing axis of the lower limb, and reduce transient lateral displacement of the shank. Therefore, dynamic control of ankle inversion–eversion during heel contact represents a rational strategy for influencing knee joint loading through distal joint alignment.

Based on this concept, the proposed insole-type assist device detects excessive heel inversion at heel contact and actively corrects it by tilting the heel outward. This correction is intended to laterally shift the COP, reduce the KAM, and consequently transfer the load-bearing line toward the lateral side of the knee joint. This study describes an insole-type assist device that provides ankle alignment correction using a compact in-shoe mechanism, without relying on bulky external actuation systems, to support lower-limb alignment during stepping-related movements. This biomechanical principle is consistent with findings reported for conventional lateral wedge insoles. For example, Yılmaz et al. demonstrated that increasing the wedge thickness of lateral insoles significantly shifted the load-bearing line laterally and reduced medial knee loading [10]. Furthermore, Ferreira et al. reported that even small variations in wedge angle can induce meaningful biomechanical changes throughout the lower limb, indicating that lateral wedges are highly sensitive in modulating load-bearing line position and KAM [11]. Unlike conventional LWIs that provide constant static correction throughout stance, the proposed device applies selective and dynamic assistance only at the moment of heel contact, when lateral thrust is most likely to occur. By limiting correction to this critical timing, the device aims to reduce overcorrection and discomfort while effectively targeting transient malalignment associated with knee OA.

Therefore, this study was designed as a feasibility study to determine whether an insole-type active assist intervention could be safely delivered and evaluated during an on-the-spot stepping task in patients with medial knee osteoarthritis, and whether motion-capture-based assessment of lateral knee thrust could be successfully conducted to inform the design of future definitive studies.

## Materials and methods

### Study design

This study was designed as a feasibility study to examine whether an insole-type active assist device could be safely delivered and evaluated during an on-the-spot stepping task in patients with medial knee osteoarthritis (OA), and whether motion-capture-based outcome assessment could be successfully conducted. This study was not intended to evaluate clinical or biomechanical effectiveness, but to inform the design of future definitive studies.

### Participants

Six ambulatory patients with medial knee OA participated in this study. All participants were diagnosed by a board-certified orthopedic surgeon based on clinical examination and standard weight-bearing radiographic assessment. Radiographic severity was classified according to the Kellgren–Lawrence grading system (Grade II–III), with four participants classified as Grade II and two as Grade III. To minimize measurement error and inter-rater variability, all radiographic evaluations were performed by the same orthopedic physician using routine clinical procedures.

All participants were confirmed by an orthopedic physician to have no severe bilateral deformity or significant difference in range of motion between the left and right knees. Individuals who had difficulty performing the stepping task or were judged by a physician to be unsuitable for participation were excluded. All six participants provided fully informed written consent, and all experimental procedures were approved by the Ethics Review Committee for Studies Involving Human Subjects at Gifu University School of Medicine (approval No. 2023 − 197).

### Insole-type assist device

**Fig. 1:**
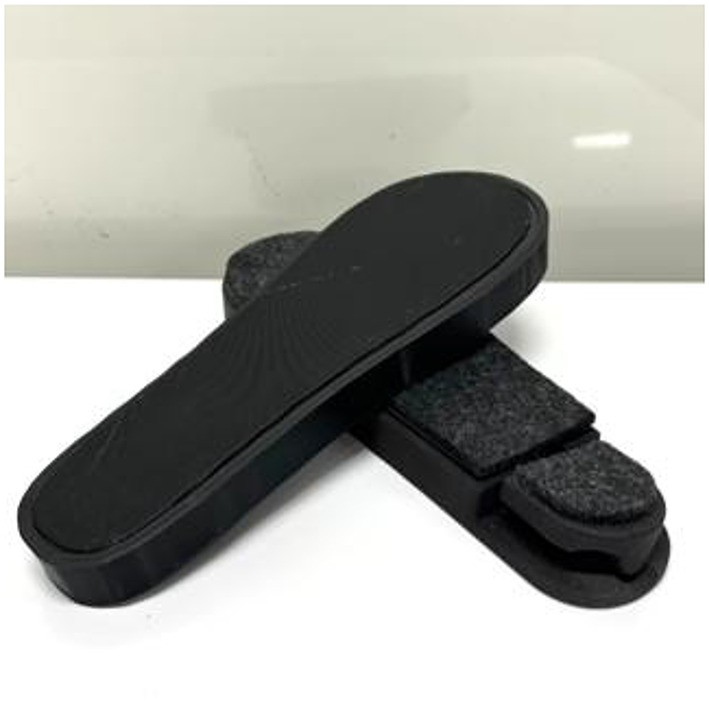
The novel insole-type active assist device for ankle alignment correction during stepping in patients with knee osteoarthritis

As illustrated in Fig. 1, the newly developed device (with a thickness of 22 mm) is designed to be worn inside the shoe, similar to an insole. The primary feature of the device is its capability to tilt the heel section laterally in the frontal plane. The device is controlled by an Arduino Nano microcontroller board and incorporates a six-axis inertial sensor (MPU-6050, InvenSense Co., San Jose, CA, USA) and a stepping motor (PKP213D05A, Oriental Motor Co., Tokyo). The entire assembly is manufactured using a 3D printer. Further device details have been reported in previous studies [12-16]. The functional specifications are summarized in Table 1.

**Table 1:** The functional specifications of the insole-type device

Item	Specifications
Weight	500 g
Size	Females: 23.5 cm, Thickness: 22 mm; Males: 27 cm, Thickness: 22 mm
Correction Angle	0° to 10° (Eversion correction from inversion to neutral)
Function	Rotation of 0° to 10° within 500ms
Durability	Endure a 75 kg × 1.2 load applied 20 mm from the center axis of the heel, 300 cycles

### Experimental procedure

The developed insole-type device was attached to the right foot, while a dummy device of identical shape and weight was attached to the left foot. The right foot was evaluated under two conditions: Active (with motor control enabled) and Inactive (without motor control). Throughout all trials, participants consistently performed the stepping task while wearing the proposed device.

To control for footwear-related confounding effects, the shoe type worn during testing was standardized across participants. All participants wore the same model of shoes provided by the investigators; shoe size was adjusted individually to ensure appropriate fit.

The experimental task was designed as an on-the-spot stepping motion rather than continuous walking to ensure participant safety and to accommodate the wired prototype configuration. A physician manually activated the switch approximately 500 ms before heel contact during each stepping motion after sufficient practice.

### Motion capture and outcome measurement

Three-dimensional motion analysis was performed using the VICON motion capture system (Nexus 1.8.5, Vicon Motion Systems Ltd., UK) with a sampling frequency of 100 Hz. The coordinate system followed the standard right-handed global frame (X-axis: forward, Y-axis: vertical, Z-axis: rightward). Raw coordinate data were processed with a fourth-order Butterworth low-pass filter (cutoff frequency: 6 Hz). Reflective markers (14 mm diameter) were attached under medical supervision to the following anatomical landmarks on both sides of the body: the anterior superior iliac spines (ASIS), mid-lateral thigh, lateral knee epicondyle, mid-lateral tibia, lateral malleolus, heel, and the second metatarsal head.

**Fig. 2:**
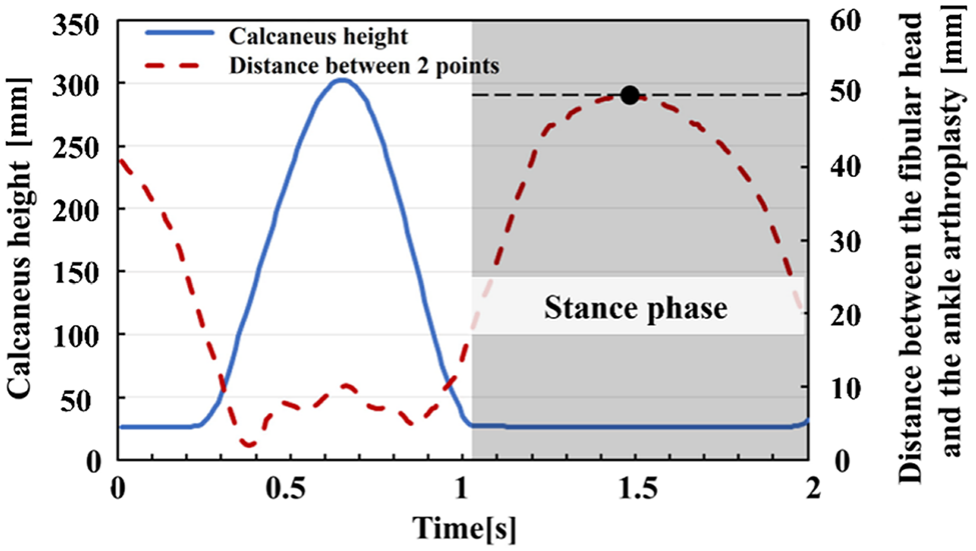
The method used to evaluate the amount of lateral thrust with the use of a motion capture system. *Red dashed line*: the distance between the fibular head and the ankle joint. *Solid blue line*: the height of the calcaneus from the ground

To quantify lateral knee thrust, the stance phase was determined from the vertical displacement of the heel marker (Fig. 2). During the stance phase, the maximum distance between the lateral knee epicondyle and lateral malleolus markers was defined as the lateral thrust value. Because using only two markers may lead to errors caused by body rotation relative to the frontal plane, pelvic rotation was calculated from the coordinates of the bilateral ASIS markers and used to correct rotational displacement between the knee and ankle joints. The corrected value was used as the effective lateral displacement (lateral knee thrust) in the frontal view, eliminating the effects of pelvic rotation and postural sway. This kinematic measure is related to the varus-thrust phenomenon, which has been described in patients with medial knee OA [17, 18]. In the present feasibility study, lateral knee thrust was used descriptively to characterize measurement variability and inform future study design.

### Feasibility outcomes and progression criteria

Feasibility outcomes were defined a priori to assess whether progression to a future definitive study would be justified. The feasibility uncertainties addressed in this study included: (1) participant recruitment and completion (2), safety of the stepping task and device use (3), activation of the assist mechanism (4), successful acquisition and analysis of motion capture data, and (5) occurrence of adverse events. Progression criteria (stop/go) were defined based on these uncertainties, including completion of the protocol by all enrolled participants, safe execution without adverse events, activation of the assist mechanism during stepping trials, complete motion capture data acquisition and analysis, and absence of device-related adverse events.

### Statistical analysis

Given the feasibility design and small sample size, only descriptive statistics were used to summarize feasibility outcomes and variability in lateral knee thrust. Inferential statistical testing was not performed. Formal power or sample size calculations were not conducted because this study was not designed to estimate effect sizes.

## Results

### Participant characteristics

Six ambulatory patients with medial knee osteoarthritis (OA) were enrolled in the study, and all participants completed the experimental protocol. The cohort consisted of two males and four females, with a mean age of 67.8 ± 7.4 years. Based on the Kellgren–Lawrence classification, four participants were classified as Grade II and two as Grade III. The mean femoro–tibial angle was 187.5° (range: 184–193°), indicating mild varus alignment. The mean body mass index was 25.5 ± 1.7 kg/m². The mean knee pain score assessed using the visual analog scale was 49.2 ± 9.1 mm, and the mean foot posture index (FPI-6) was − 3.0 ± 0.8, indicating a slightly supinated foot posture.

### Feasibility outcomes

All predefined feasibility outcomes were achieved. All six participants provided informed consent, were successfully recruited, and completed all stepping trials without withdrawal, indicating successful recruitment and retention. The on-the-spot stepping task was safely performed by all participants while wearing the insole-type assist device. No device-related adverse events, discomfort requiring termination, or safety concerns were observed during the experiments.

The assist mechanism was activated during stepping trials in five of the six participants (83%), demonstrating the technical feasibility of dynamic heel adjustment during the task. Across all participants, a total of 90 stepping trials (15 trials × 6 participants) were completed under each condition. Motion capture data were successfully acquired and processed for all trials, with no datasets excluded due to marker loss, hardware malfunction, or data-processing errors.

### Descriptive outcomes of lateral knee thrust

Lateral knee thrust was successfully quantified for all participants under both the Active (with motor control) and Inactive (without motor control) conditions. Lateral knee thrust values were first averaged across trials within each participant, and the participant-level means were then summarized descriptively at the group level.

Group-level descriptive statistics are summarized in Table 2. Substantial inter-individual variability in lateral knee thrust was observed under both conditions. The mean ± standard deviation of participant-level lateral knee thrust was 64.3 ± 24.1 mm under the Active condition and 66.1 ± 25.0 mm under the Inactive condition, with overlapping ranges between conditions (Active: 28.2–110.5 mm; Inactive: 28.0–104.3 mm).

**Table 2:** Descriptive summary of lateral knee thrust and assist activation (group level)

Item	Active condition (with control)	Inactive condition (without control)
Number of participants	6	6
Total stepping trials analyzed	90 (15 × 6)	90 (15 × 6)
Participants with at least one assist activation, n (%)	5 (83%)	–
Total assisted steps, n (%)	39 (43%)	–
Mean assisted angle during activated steps (°)	4.9 ± 2.0	–
Lateral knee thrust, mean ± SD (mm)	64.3 ± 24.1	66.1 ± 25.0

Notes: Values are presented as descriptive statistics only. No inferential statistical analyses were performed. Lateral knee thrust values represent group-level summaries based on participant-level means across stepping trials. The table is provided to illustrate measurement feasibility and variability rather than to indicate effectiveness or efficacy

Because this study was designed as a feasibility study and was not powered to evaluate effectiveness, no inferential statistical comparisons were performed. The observed variability in lateral knee thrust and assist activation provides descriptive information regarding measurement dispersion and supports the feasibility of motion-capture-based assessment in this patient population.

### Progression criteria

All predefined progression (stop/go) criteria were met. Specifically, participant recruitment and protocol completion were successful, the stepping task and device use were safe, the assist mechanism was operable during stepping trials, complete motion capture datasets were obtained for all trials, and no adverse events were observed. These findings support progression to a future larger-scale study designed to formally evaluate clinical and biomechanical outcomes.

## Discussion

The present study was designed as a feasibility study to examine whether an insole-type active assist device could be safely implemented and evaluated during an on-the-spot stepping task in patients with medial knee osteoarthritis, and whether motion-capture-based outcome assessment could be successfully conducted. Accordingly, the primary focus of this study was not to determine biomechanical or clinical effectiveness, but to assess the feasibility of the study procedures, device operation, and data acquisition in this population.

The results demonstrated that all participants were able to complete the stepping task safely while wearing the device, and that motion capture data could be consistently acquired and analyzed across repeated trials. In addition, the assist mechanism was successfully activated during stepping in several participants, indicating that the control concept could be implemented under experimental conditions. Importantly, however, substantial variability was observed across participants and stepping trials in both ankle posture at heel contact and lateral knee thrust measurements. These findings highlight the heterogeneity inherent in stepping mechanics among individuals with knee osteoarthritis and underscore the importance of feasibility testing prior to conducting definitive studies.

Because of the small sample size and feasibility-oriented design, the present study cannot draw conclusions regarding the biomechanical effectiveness of dynamic ankle alignment correction or its influence on medial knee loading. Observed variations in lateral knee thrust across conditions should therefore be interpreted solely as descriptive information to inform future study design, rather than as evidence of an intervention effect. In particular, statements regarding confirmation of ankle posture at heel contact or reduction of knee loading are beyond the scope of this feasibility study and are not supported by the present data.

The variability observed in assist activation and kinematic outcomes suggests that further refinement of the control strategy, including automated and sensor-driven triggering of assistance, may be necessary to ensure consistent device engagement during gait-related tasks. These feasibility findings provide practical insights into protocol design, outcome selection, and sample size considerations for future adequately powered studies, in which clinical and biomechanical effectiveness can be rigorously evaluated.

## Conclusion

This feasibility study demonstrated that an insole-type active assist device can be safely implemented during an on-the-spot stepping task in patients with medial knee osteoarthritis, and that motion-capture-based assessment of lower-limb kinematics can be successfully conducted in this context. All participants completed the experimental protocol without adverse events, and the assist mechanism and data acquisition procedures were operable under controlled experimental conditions.

Importantly, this study was not designed to evaluate biomechanical or clinical effectiveness. The findings are therefore limited to feasibility-related outcomes and descriptive characterization of measurement variability. These results provide practical information to support the design of future, adequately powered studies in which the effectiveness of dynamic ankle alignment correction during gait can be rigorously evaluated.

## Data Availability

The datasets used and/or analyzed during the current study are available from the corresponding author on reasonable request.

## References

[1] Murphy L, Schwartz AT, Helmick GC, Renner BJ, Tudor G, Koch G, Dragomir A, Kalsbeek DW, Luta G, Jordan MJ. Lifetime risk of symptomatic knee osteoarthritis. Arthritis Care \& Research. 2008;59(9):1207–1213. 10.1002/art.24021 PMC4516049 18759314

[2] Hayashi N, Aoki T. Orthopedic exercise therapy navigation based on joint functional anatomy[Lower Limb]. MEDICALVIEW CO., LTD. 2014;112–115.

[3] Takeda M, Sato K, Hirata Y, Katayama T, Mizuta Y, Koujina A. Standing, walking, and sitting support robot based on user state Estimation. IEEE Access. 2021;9:152677–87.

[4] Ugurlu B, Oshima H, Sariyildiz E, Narikiyo T, Babic J. Active compliance control reduces upper body effort in exoskeleton-supported walking. IEEE Trans Human-Machine Syst. 2020;50(2):144–53.

[5] Tsukahara A, Hasegawa Y, Eguchi K, Sankai Y. Restoration of gait for spinal cord injury patients using HAL with intention estimator for preferable swing speed. IEEE Trans Neural Syst Rehabilitation Eng. 2014;23(2):308–18. 10.1109/TNSRE.2014.2364618 25350933

[6] Haida N. Cell science of disuse muscular atrophy. Japan Soc Phys Therapy. 1994;21(2):94–7.

[7] Du W, Guo Y, Wang C, Cui W, Chen W, Li X. Biomechanical response of lower limb joints to lateral wedge insoles. Sci Rep. 2024;14(107). 10.1038/s41598-023-50693-1. 10.1038/s41598-023-50693-1 PMC10762160 38167577

[8] Hinman SR, Bowles AK, Bennell LK. Laterally wedged insoles in knee osteoarthritis: do Biomechanical effects decline after one month of wear? BMC Musculoskelet Disord. 2018;10:146. 10.1186/1471-2474-10-146 PMC2791095 19939281

[9] Neumann DA. Kinesiology of the musculoskeletal system foundationas for rehabilitation. Mosby. 2012.

[10] Yılmaz B, Kesikburun S, Köroğlu O, Yaşar E, Göktepe AS, Yazıcıoğlu K. Effects of two different degrees of lateral-wedge insoles on unilateral lower extremity load-bearing line in patients with medial knee osteoarthritis. Acta Orthop Traumatol Turc. 2016;50(4):405–8. 27452743 10.1016/j.aott.2016.06.004 PMC6197552

[11] Ferreira V, Machado L, Vilaça A, Xará-Leite F, Roriz P. Can slight variations to lateral wedge insoles induce significant Biomechanical changes in patients with knee osteoarthritis? Biomechanics. 2022;2(3):342–51.

[12] Chang A, Hayes K, Dunlop D, Hurwitz D, Song J, Cahue S, Genge R, Sharma L. Thrust during ambulation and the progression of knee osteoarthritis. Arthritis Rheum. 2004;50(12):3897–903. 15593195 10.1002/art.20657

[13] Kuroyanagi Y, Nagura T, Kiriyama Y, Matsumoto H, Otani T, Toyama Y, Suda Y. A quantitative assessment of varus thrust in patients with medial knee osteoarthritis. Knee. 2012;19(2):130–4. 21300549 10.1016/j.knee.2010.12.007

[14] Itami T, Date K, Ishii Y, Yoneyama J, Aoki T. Insole-type walking assist device capable of inducing inversion-eversion of the ankle angle to the neutral position, 2023 IEEE/RSJ International Conference on Intelligent Robots and Systems (IROS). 2023.

[15] Ishii Y, Date K, Itami T, Yoneyama J, Matsui N, Shinoda N, Aoki T. Shoe-type walking assist device with controllable subtalar joint alignment at heel contact, 2023 International Conference on Rehabilitation Robotics (ICORR). 2023. 10.1109/ICORR58425.2023.10304713 37941187

[16] Ishii Y, Date K, Itami T, Yoneyama J, Matsui N, Shinoda N, Aoki T. Shoe type walking assist robot orthosis capable of controlling subtalar joint Inversion/Eversion angle at heel contact. J Japanese Soc Musculoskelet Med. 2023;34(3):279–87.

[17] Yoshihara M, Ishii Y, Itami T, Yoneyama J, Aoki T. Effects of Insole-Type Device with Controllable Ankle Joint Angle on the Peroneus Longus Muscle During Foot Stomping Motion, IEEE RAS EMBS 10th International Conference on Biomedical Robotics and Biomechatronics (BioRob 2024). 2024.

[18] Hirota R, Ishii Y, Yoshihara M, Itami T, Iwase M, Oi Y. Insole-type walking support device equipped with a control method to eliminate rattling, 2024 IEEE-RAS 23rd International Conference on Humanoid Robots (Humanoids). 2024;655–660.

